# *Clostridioides difficile* infection in infants: a case report and literature review

**DOI:** 10.1186/s13099-023-00552-1

**Published:** 2023-06-29

**Authors:** Zhirong Li, Ning Dong, Jihong Hao, Zirou Ouyang, Cuixin Qiang, Ying Yang, Chaoyi Mi, Yanan Niu, Jing Yang, Baojiang Wen, Liwei Wang, Shaodan Zhang, Jianhong Zhao

**Affiliations:** 1 Hebei Provincial Center for Clinical Laboratories, The Second Hospital of Hebei Medical University, Shijiazhuang, 050000 Hebei China; 2 Department of Clinical Laboratory, The Second Hospital of Hebei Medical University, Shijiazhuang, 050000 Hebei China; 3 Research Center, The Fourth Hospital of Hebei Medical University, Shijiazhuang, 050011 Hebei China; 4 Department of Pediatrics, The Second Hospital of Hebei Medical University, Shijiazhuang, 050000 Hebei China; 5 Clinical Laboratory, Shexian Hospital, Handan, 050000 Hebei China; 6 215# Hepingxi road, Shijiazhuang, Hebei province China

**Keywords:** *Clostridioides difficile*, CDI, Antibiotic-associated diarrhea, Intestinal microbiota, Infant

## Abstract

**Background:**

*Clostridioides difficile* (*C. difficile*) is the major pathogen causing antibiotic-associated diarrhea. There are a variety of symptoms associated with *C. difficile* infection (CDI) in adults, including self-limiting diarrhea, pseudomembranous colitis, toxic megacolon, septic shock, and even death from the infection. However, the infant’s intestine appears to be completely resistant to the effects of *C. difficile* toxins A and B with rare development of clinical symptoms.

**Case presentation:**

In this study, we reported a 1-month-old girl with CDI who was born with neonatal hypoglycemia and necrotizing enterocolitis. Her symptom of diarrhea occurred after extensive use of broad-spectrum antibiotics during hospitalization and was accompanied by elevated white blood cell, platelet, and C-reactive protein levels, and repeated routine stool examinations were abnormal. She was recovered by norvancomycin (an analogue of vancomycin) and probiotic treatment. The results of 16 S rRNA gene sequencing also demonstrated the recovery of intestinal microbiota with the enrichment of *Firmicutes* and *Lactobacillus*.

**Conclusions:**

Based on the literature review and this case report, clinicians should also pay attention to diarrhea caused by *C. difficile* in infants and young children. More strong evidence is needed to explain the true prevalence of CDI in this population and to better understand the *C. difficile*-associated diarrhea in infants.

**Supplementary Information:**

The online version contains supplementary material available at 10.1186/s13099-023-00552-1.

## Introduction

*Clostridioides difficile* (*C. difficile*) is a Gram-positive anaerobic bacillus that is a major pathogen causing healthcare-associated infections [1]. Since the early 21st century, *Clostridioides difficile* infection (CDI) has been a major global public health problem, and is considered an “urgent threat” to human health by the United States Centers for Disease Control and Prevention [2]. *C. difficile* pathogenicity is mediated by the protein toxins A and B (encoded by *tcdA* and *tcdB*, respectively), which cause clinical symptoms ranging from self-limited diarrhea to life-threatening pseudomembrous colitis, toxic megacolon, and even death [3, 4]. The burden of this disease has increased over the past few decades, especially outbreaks of the hypervirulent strain RT027/NAP1/BI, increasing the morbidity and mortality of hospital-acquired infections worldwide [5]. It is estimated that CDI is responsible for over 500,000 enteric infections, 29,000 deaths and over $4.8 billion in healthcare costs each year in the United States [6]. In addition, CDI is no longer restricted to the hospital setting, and higher rates have been reported in traditionally low-risk populations, including children who have not been exposed to hospital settings [7].

A CDI treatment plan depends on whether it is an initial or recurrent episode, as well as its severity [8]. The first episode of non-severe or severe CDI can be treated with vancomycin or fidaxomicin. For fulminant CDI, vancomycin is the treatment of choice. In the event of ileus, vancomycin may also be administered through the rectum. Especially if an ileus is present, it is recommended to administer oral or rectal vancomycin with intravenous metronidazole. The first recurrence of CDI can be treated with oral vancomycin or fidaxomicin for 10 days, or a prolonged taper of pulse oral vancomycin. CDI patients with a second or subsequent recurrence can be treated with oral vancomycin therapy using a tapered and pulsed regimen, fidaxomicin, and vancomycin followed by Rifaximin or fecal microbiota transplantation (FMT).

It is thought that CDI develops because of an imbalance in the host intestinal microbiota, which can be caused by a variety of factors. Broad-spectrum antimicrobials are considered to be the most important risk factor for CDI in adults and children by destroying the host intestinal microbiota and reducing colonization resistance to *C. difficile* and other enteric pathogens [9]. Earlier studies have shown a marked reduction in bacterial diversity among subjects with CDI. CDI patients were enriched with *Enterococcus*, *Enterobacteriaceae*, *Erysipelotrichaceae*, and *Gammaproteobacteria* class, but there was a decrease in *Ruminococcaceae*, *Lachnospiraceae*, *Bacteroidetes*, and *Clostridial clusters IV* and *XIVa* [10].

The epidemiology of *C. difficile* in children is characterized by asymptomatic colonization in many infants, with the highest colonization rates (which can exceed 40%) particularly among infants younger than 12 months of age [11]. Thus, the consensus guidelines recommend testing for *C. difficile* only if infants (< 12 months of age) present with pseudomembranous colitis or toxic megacolon or if they have symptoms of clinically significant diarrhea in which other causes of diarrhea have been ruled out [8]. However, a study based on children with diarrhea in Kenya showed that children with diarrhea were more susceptible to *C. difficile* infection than children with rotavirus or *Cryptosporidium* infection [12]. Here, we describe the clinical manifestations, diagnosis, treatment, changes in the intestinal microbiota of a pediatric patient with CDI, and reviewed the literature to improve clinicians’ awareness of CDI.

### Case presentation

#### Clinical presentation

The pediatric patient was a 1-month-old female who came to the outpatient department for the first time due to diarrhea (7–8 loose yellow stools/day) more than 20 days ago. Diarrhea continued after treatment with cefixime and probiotics (*Saccharomyces boulardii*). She was admitted to our hospital on March 4, 2021. The infant was born by caesarean section at full term and was diagnosed with hypoglycemia and necrotizing enterocolitis (NEC). She was treated with broad-spectrum antibiotics during hospitalization, including cefoperazone, meropenem, piperacillin, tazobactam, sulbactam, fluconazole, vancomycin, and azithromycin.

#### Clinical findings

Clinical examination on admission revealed: temperature, 36.8℃; heart rate, 136 beats/min; and respiratory rate, 35 breaths/min. The infant was conscious and presented pharyngeal hyperemia, mildly swollen tonsils, and coarse breath sounds. The patient did not have fine wet rales and lower limb edema.

#### Diagnostic focus and assessment

Laboratory tests performed on the day of admission revealed the following results: white blood cell count, 26.3 × 10^9^/L; neutrophil count, 11.47 × 10^9^/L; lymphocyte count, 6.87 × 10^9^/L; red blood cell count, 3.36 × 10^12^/L; hemoglobin, 104 g/L; platelet count, 599 × 10^9^/L; C-reactive protein (CRP), 157.2 mg/L; albumin, 31.4 g/L; lactate dehydrogenase, 355.0 U/L; and procalcitonin, 0.20 ng/ml. Stool routine examination showed a fecal white blood cell count of 99 − 120 per high power field (HPF) and fecal red blood cell count of 15 − 25/HPF (Supplementary Table S1). Bone marrow aspiration revealed granulocytosis and thrombocytosis and a granulocytic-to-erythroid ratio of 23%. Stool cultures revealed *Enterococci* as the main bacteria, with intestinal parasites, rotavirus, and common bacterial intestinal pathogens (*Shigella*, *Salmonella*, Pathogenic *Escherichia coli*, etc.) remaining undetected.

After admission, she was initially diagnosed with neonatal acute diarrhea according to the Textbook of Pediatrics (9th edition, People’s Health Publishing House, 2018) [13]. In this case, a laboratory test revealed elevated white blood cell count and CRP in peripheral blood, bloody purulent stool, and a routine stool examination revealed increased white and red blood cells. After excluding the above intestinal pathogens, the pathogen of this case was considered to be invasive bacteria, and empirical treatment was performed with cefmenoxime and norvancomycin (an analogue of vancomycin). After 3 days of treatment, the frequency of diarrhea decreased to about 4 times/day.

Given the patient’s long-term history of antibiotic use, *C. difficile* related tests were performed on the 3rd and 7th day of admission. As a result, enzyme immunoassay (EIA) for *C. difficile* glutamate dehydrogenase (GDH) and nucleic acid amplification tests (NAATs) for toxin genes were positive. Bacterial culture revealed colonies of *C. difficile*, which were confirmed to be sequence typing 54 by multilocus sequence typing. Based on the above test results, the patient was finally diagnosed with CDI, so cefmenoxime was discontinued and norvancomycin was continued.

#### Therapeutic focus and assessment

After we treated the patient with norvancomycin for 12 days, the infant stool frequency decreased, about 2–3 times/day, and the stool was sticky, with no fever, nausea, or abdominal pain, and was discharged. After discharge, intravenous antibiotics were discontinued and changed to a combination of oral norvancomycin and probiotics.

**Fig. 1:**
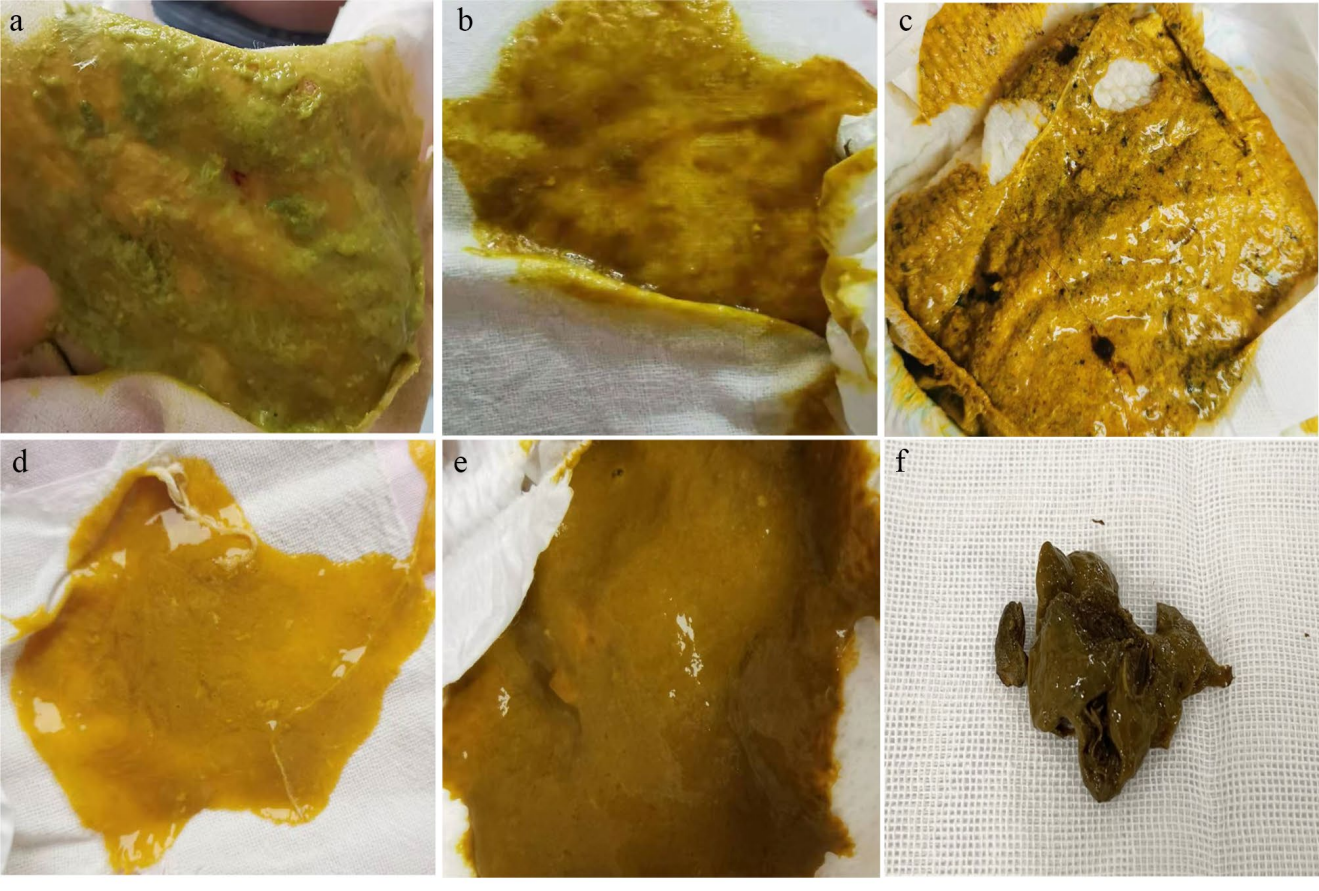
Stool characteristics during treatment. **a** Viscous stool after the first course of treatment; **b** Loose and yellow stool after treatment withdrawal; **c** Mushy stool after the second course of treatment; **d** Loose and yellow stool after treatment withdrawal; **e** Normal stool after the third course of treatment; **f** Normal stool during the 3-month follow-up

**Fig. 2:**
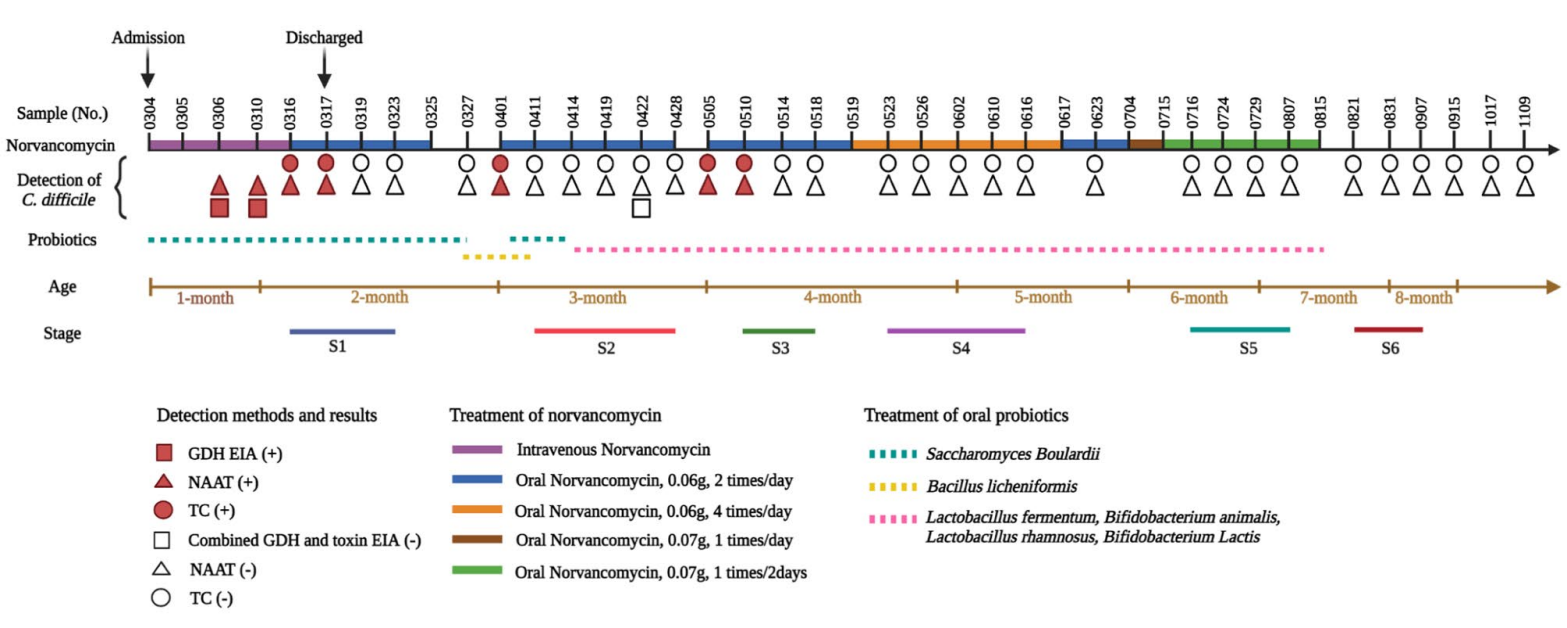
Diagnosis and treatment of *Clostridioides difficile* infection. Samples were numbered according to the date of collection. The treatment cycle was divided into six stages (S1 to S6) according to sampling time and treatment dose. S1 to S5 correspond to different treatment doses, and S6 is the recovery stage

The patient was treated with three courses of antibiotics with oral norvancomycin after hospital discharge. After the first course (9 days) of treatment, the infant’s stools were viscous (Fig. 1a) with a frequency of about 3 times/day, and routine stool examination shows normal white and red blood cell counts (Supplementary Table S1). However, 7 days after treatment withdrawal, the patient began to have frequent loose stools (Fig. 1b), about 6 times/day. The routine stool examination showed elevated white blood cell count (Supplementary Table S1). *C. difficile* toxigenic culture (TC) and NAATs were positive. The second course of antibiotics was started based on symptoms and test results. After 25 days of treatment, the patient’s routine stool examination and frequency returned to normal (2–3 times/day) (Supplementary Table S1). The stool was mushy (Fig. 1c), and stool culture and NAATs were negative. Ten days after treatment discontinuation, the patient began to have frequent loose, mucus-containing stool (Fig. 1d), approximately 5 times/day. The routine stool examination showed fecal white blood cell count of 26–44/HPF (Supplementary Table S1), TC and NAATs were positive. The treatment period was extended based on clinical symptoms and the recurrence of diarrhea. In the third course, the dose of norvancomycin was adjusted five times sequentially according to clinical status as follows: 0.06 g twice a day for 10 days, 0.06 g four times a day for 29 days, 0.06 g twice a day for 17 days, 0.07 g once a day for 11 days, and 0.07 g once every 2 days for 31 days. Stool frequency returned to normal after the third course of treatment, about 2 times/day, the stool was normal (Supplementary Table S1), stool culture of *C. difficile*, GDH/toxin EIA, and NAAT were negative (Fig. 1e and f). The diagnosis and treatment details are shown in Fig. 2.

### 16 S rRNA gene-based fecal microbiota profiling

**Fig. 3:**
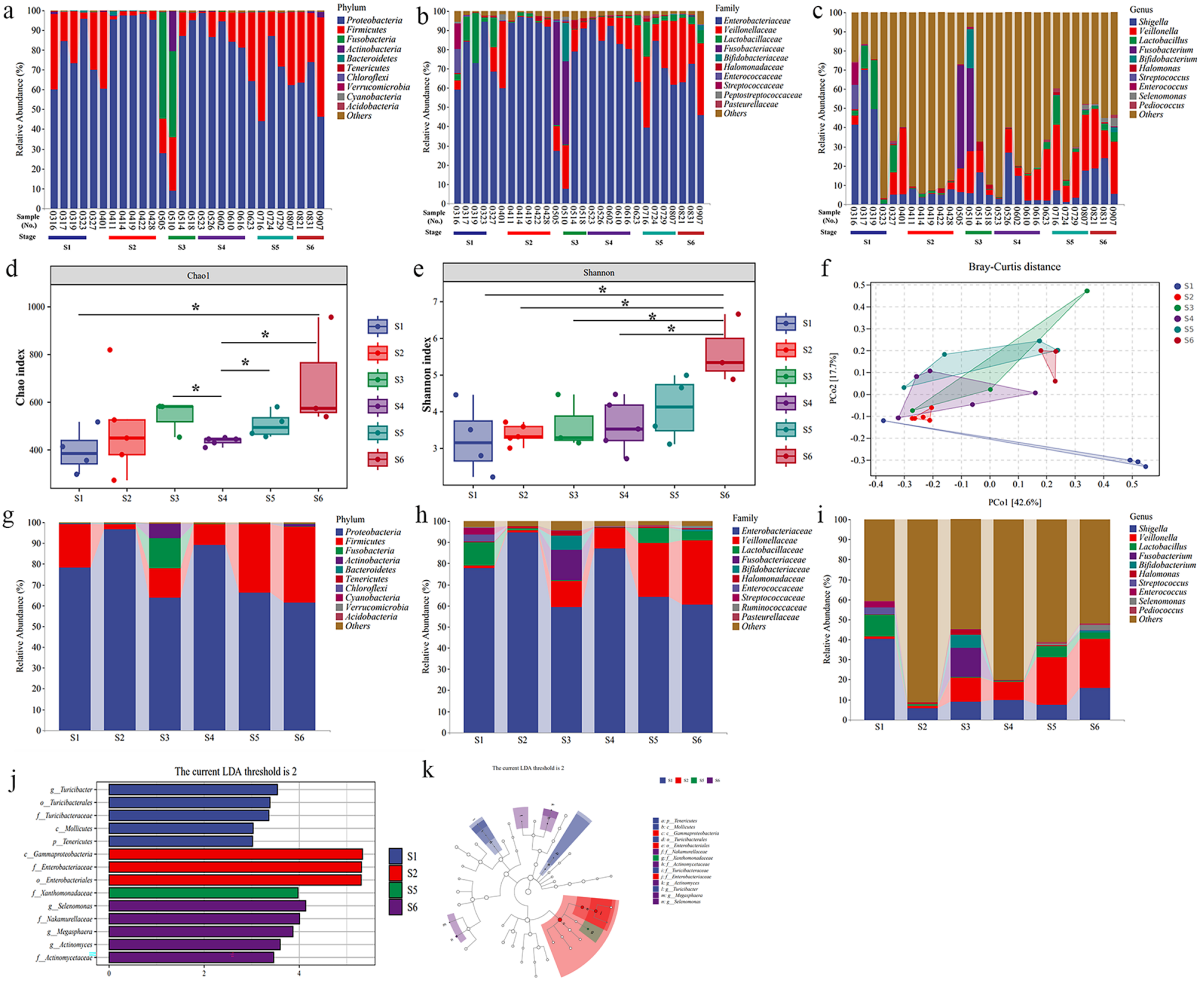
Composition, abundance, diversity, and predominance of intestinal microorganisms in our patient. **a** Phylum-level taxonomic distribution of the microbial community; **b** Family-level taxonomic distribution of the microbial community; **c** Genue-level taxonomic distribution of the microbial community; **d-e** Alpha diversity analysis (Chao and Shannon index) of metagenomic sequencing data (≥ 3 samples per stage). Data are mean ± SEM. Differences between data were assessed using the Kruskal-Wallis test and Dunn’s test. ^*^P < 0.05; **f** Principal coordinate analysis of metagenomic sequencing data at different stages (≥ 3 samples per stage); **g-i** Relative abundance of bacterial phyla (g), families (h) and genus (i) at different stages; **j-k** Predominant microorganisms across stages (≥ 3 samples per stage). (j) Hierarchical tree diagram based on linear discriminant analysis effect size; (k) Distribution histogram based on linear discriminant analysis

To assess changes in the fecal microbiota during treatment, the treatment course was divided into six stages (S1 to S6) (≥ 3 stool samples per stage) according to sampling time and treatment dose (Figs. 2 and 3). S1 to S5 corresponded to different treatment doses, and S6 was the recovery stage. Samples were collected in a sterile collection tube, and stored at -80℃ until analysis.

The OMEGA Soil DNA Kit (M5635-02) (OMEGA Bio-Tek, Norcross, GA, USA) was used to extract total genomic DNA from stool samples. The fecal microbiota characteristics were determined by sequencing the V3-V4 region of the 16 S rRNA gene using the Illlumina NovaSeq platform with NovaSeq 6000 SP Reagent Kit (500 cycles) at Shanghai Personal Biotechnology Co., Ltd (Shanghai, China).

QIIME2 2019.4 was used for microbiome bioinformatics with slight modifications in accordance with the official tutorials (https://docs.qiime2.org/2019.4/tutorials/). Sequence data were demultiplexed using the demux plugin, followed by primers being cut with the cutadapt plugin. The DADA2 plugin then filtered, denoised, merged, and removed chimera from sequences [14]. An amplicon sequence variant (ASV)’s taxonomy was assigned using the classify-sklearn Naive Bayes classifier (Greengenes Database) in the feature-classifier plugin [15]. In order to obtain the sharing information between groups, the relative abundance of ASVs and Venn diagram was analyzed. Each group’s Alpha diversity level was then calculated based on the distribution of ASVs. In addition, to measure the difference in beta diversity between each group, the distance matrix for each sample was calculated, and the principal coordinate analysis (PCoA) was performed.

Composition analysis of the fecal microbiota of each sample is shown in Fig. 3a and c. Alpha diversity (Chao and Shannon indexes) was significantly higher at S6 than at S1, S2, S3, and S4 (*P* < 0.05, Fig. 3d and e). PCoA based on the Bray-Curtis distance showed a separation in the fecal microbiota structure at S5 and S6 relative to S2 (*P* < 0.005, Fig. 3f), indicating that the microbiota was significantly impacted by treatment. At the phylum level, *Firmicutes* was enriched at S5 and S6 (Fig. 3g). At the family level, *Veillonellaceae* and *Lactobacillaceae* were enriched (Fig. 3h). At the genus level, *Veillonella* and *Lactobacillus* were enriched (Fig. 3i). Bacterial phylotypes were identified at each stage using linear discriminant analysis effect size. The results showed no significant differences in phylotypes across stages (Fig. 3j and k). Fecal microbiota composition reflected by alpha and beta diversity changed over time, possibly due to CDI.

### Follow-up and outcomes

Following three courses of norvancomycin treatment, stool frequency returned to normal, and diarrhea did not recur during three months of follow-up.

## Discussion and conclusions

Early childhood is a crucial period during which the intestinal microbiota may impact current and future health status [16]. In adults, *C. difficile* primarily colonizes the lower intestinal tract and causes colonic inflammation by binding to toxins A and B to receptors on the plasma membrane [17, 18]. However, children’s intestines appear to be resistant to the effects of these toxins, and clinical infections are rare. *C. difficile* has been recovered from an average of 37% of stools in healthy infants younger than 1 month. The colonization rate decreases to approximately 30% between 1 and 6 months of age. During the first year of life, this rate declines until it reaches 10% in healthy infants. At 3 years old, the colonization rate is approximately 3%, similar to the adult carrier rate [19–21]. Neonatal resistance to CDI may be related to the absence of toxin receptors, downstream signaling pathways in the immature intestinal mucosa, and some protective factors in breast milk and the host intestinal microbiota [22, 23]. Therefore, the consensus guidelines recommend clinical testing for CDI only in the presence of clinical indications [8]. In recent studies, 26% of children hospitalized with CDI were younger than 1 year of age, and 5% were newborns [24]. Pediatric CDI is caused by a variety of factors, including age, gender, comorbidities, prolonged hospitalization and enteral feeding, but antibiotic exposure appears to be the main risk factor [25]. In spite of the fact that nearly all antibiotic classes can cause CDI, clindamycin, cephalosporins, and fluoroquinolones appear to pose the greatest threat [26]. Broad-spectrum antibiotic treatment can damage the host intestinal microbiota and reduce intestinal commensal flora diversity and beneficial bacteria abundance, leading to excessive growth and toxin production of *C. difficile* [27, 28]. Following the discontinuation of antibiotic treatment, these effects can persist for weeks or months, predisposing individuals to *C. difficile* infections.[29].

There is a wide range of clinical manifestations of CDI. It is possible for patients with a mild infection to have a single episode of diarrhea, as well as abdominal pain, bloody stools, purulent stools, or watery stools. In severe cases, patients may develop pseudomembranous colitis, fulminant colitis, toxic megacolon, and sepsis [4]. In this study, we searched Wanfang Data, China National Knowledge Infrastructure, and the Biomedical Literature Database (PubMed) for case reports on CDI in children. Studies that did not exclude other pathogens and had unclear detection methods related to *C. difficile* were excluded. A summary of the national and international studies is listed in Table 1 [30–40]. Combined with the literature review, the main clinical manifestations of CDI in children are fever, diarrhea, abdominal pain, vomiting and pseudomembranous colitis, and some children have rare clinical manifestations such as reactive arthritis. Increased white blood cell and CRP counts were found in laboratory tests. Our patient was treated with broad-spectrum antibiotics during hospitalization for hypoglycemia and NEC at birth. In the early stage of the onset, the main manifestations were diarrhea (loose yellow stool), repeated routine stool examinations were abnormal, and toxigenic *C. difficile*, GDH, and NAATs were positive. After excluding other causes, the patient was finally diagnosed with CDI.

**Table 1:** Cases of *Clostridioides difficile* infection in children

Author	Year	Age	Sex	Exposure	Symptoms	Laboratory results	Other pathogens	Comorbidities	Detection of *C. difficile*	Treatment	Outcome
Cappella M et al.[30]	2016	6-year	Male	Amoxicillin-clavulanate	Watery diarrhea, abdominal pains, fever, reactive Arthritis	CRP, mg/L: 39 Erythrocyte sedimentation rate, mm/h: 30	*Salmonella* (-); *Shigella* (-); *Yersinia* (-); *Campylobacter* (-); Viruses (-)	No	ELISA (Toxins)	Oral naproxen and metronidazole	Cured
Durand CL et al.[31]	2009	10-year	Female	Erythromycin; Penicillin	Hip pain, diarrhea, fever, reactive Arthritis	CRP, mg/dL: 12 WBC, 10^9^/L: 15.4	Bacteria (-); Viruses (-); Ova (-); Cysts (-); Parasites (-)	No	ELISA (Toxins)	Oral and intravenous metronidazole	Cured
Liang Y et al.[32]	2020	6-year	Female	Chemotherapy; Cephalosporin; Vancomycin; Imipenem; Carprofen	Fever, diarrhea with jelly-like stools, abdominal distension, nausea, vomiting, shortness of breath, massive hydrothorax and ascites	CRP, mg/L: 220 WBC, 10^9^/L: 15.4 Hemoglobin, g/dL: 6.9 Albumin, g/dL: 2.8 Dehydrogenase, U/L: 282	Common bacteria and fungiculture (-); Rotavirus (-); Adenoviridae antigens (-)	Lymphoma	RT-PCR (Toxin gene)	Oral vancomycin and *Saccharomyces boulardii*	Cured
Rojas GM et al.[33]	2018	5-month	Female	No	Watery diarrhea, abdominal distension	WBC, 10^3^/µL: 9.5 CRP, mg/dL: 18 Erythrocyte sedimentation rate, mm/h: 64	*Salmonella* (-); *Shigella* (-); *E. coli* O157:H7 (-); *Vibrio* (-); *Yersinia* (-); *Campylobacter* (-)	Kawasaki disease	PCR (Toxin gene)	Oral metronidazole	NA
Rojas GM et al.[33]	2018	4-year	Male	Penicillin	Non-bilious, non-bloody vomiting and abdominal distension	WBC, 10^3^/µL: 9.2 CRP, mg/dL: 17.6 Erythrocyte sedimentation rate, mm/h: 110	*Salmonella* (-); *Shigella* (-); *E. coli* O157:H7 (-); *Vibrio* (-); *Yersinia* (-); *Campylobacter* (-)	Kawasaki disease	PCR (Toxin gene)	Oral metronidazole	NA
Price EH et al.[34]	1988	2-month	Female	No	Non-bloody diarrhea, vomiting, fever, acute enterocolitis	WBC, 10^9^/L: 4.4 PLT, 10^9^/L: 305–616	*Campylobacter* (-); enteropathogenic *Escherichia coli* (-); *Salmonella* (-); *Shigella* (-); Viruses (-); *E coli* 013 (a non-enteropathogenic serotype) (+); *Yersinia enterocolitica* (-); Pseudotuberculosis (-)	No	TC	Intravenous metronidazole	Cured
Price EH et al.[34]	1988	3-week	Female	Anticholinergic drug (pipenzolate bromide)	Profuse watery, but non-bloody diarrhea, acute enterocolitis	WBC, 10^9^/L: 20.5 PLT, 10^9^/L: 25,000	enteropathogenic *E coli* (-); *Salmonella* (-); *Shigella* (-); *Campylobacter* (-); Viruses (-); *Yersinia enterocolitica* (-)	No	TC	Intravenous benzylpenicillin, gentamicin and metronidazole	Cured
Loffler HA et al.[35]	2004	6-year	Female	NA	Fever, nausea, vomit, watery diarrhea, reactive arthritis	WBC, 10^9^/L: 16.9 CRP, mg/L: 21 Erythrocyte sedimentation rate: 61 mm/h Fibrinogen, g/L: 9	*Yersinia enterocolitica* (-); *Shigella* (-); *Salmonella* (-); *Campylobacter* (-)	No	EIA (Toxins)	Diclofenac and vancomycin	Cured
Nogueira H et al.[36]	2021	2.5-year	Female	Immunosuppressive medication (Prednisone/Tacrolimus/Mycophenolate sodium)	Bloody and mucoid stool	WBC, /mm3: 10,420 Hemoglobin, g/dL: 10.5	Intestinal parasites (i.e. protozoa and helminths) (-); Gastrointestinal viruses (-); Enteric pathogens (-)	Liver transplantation	TC; EIA (Toxins)	Metronidazole	Cured
Quesada-Gomez C et al.[37]	2012	18-month	Female	Azithromycin; Diclofenac	Watery diarrhea with mucous	Normal	Protozoa (-); Helminthes (-); Gastrointestinal viruses (-); Bacterial enteric pathogens (-)	No	TC, EIA (Toxins)	Metronidazole and probiotic (commercial Saccharomyces and Bacillus spores)	Cured
Yang Hongbin et al.[38]	2018	2-year	Female	Cephalosporin	Diarrhea	WBC, 10^9^/L: 14.63 PLT, 10^9^/L: 539	Mycobacterium tuberculosis (-); Cytomegalovirus (-); Herpes simplex virus (-)	No	Colonoscopy; EIA (GDH/Toxins)	Oral metronidazole and probiotics (Bacillus subtilis)	Cured
Guan Jun et al.[39]	2017	6-year	Female	Cefadroxil; Cephalosporins; Cefixime	Diarrhea, abdominal pain	ALT:9.0 IU/L AST: 30I U/L Alpha hydroxybutyrate dehydrogenase, IU/L: 455 Lactic dehydrogenase, IU/L: 509	*Shigella* (-); *Salmonella* (-); Pathogenic *Escherichia coli* (-); Vibrio cholerae (-); Campylobacter jejuni (-); Rotavirus (-); Enterovirus (-); Cytomegalovirus (-); EB virus (-)	No	Colonoscopy; EIA (GDH/Toxins)	Oral metronidazole and probiotics (Saccharomyces boulardii)	Cured
Kader A et al.[40]	2004	7-week	Male	No	Loose stools and faltering weight	WBC, 10^9^/L: 21.05 CRP, mg/L: 45	Bacteria (-); Viruses (-); Ova (-); Cysts (-)	No	ELISA (Toxins)	Oral metronidazole	Cured
Our patient	2021	1-month	Female	Piperacillin, tazobactam, cefoperazone, sulbactam, fluconazole, vancomycin meropenem and azithromycin	Diarrhea, loose yellow stools	WBC, 10^9^/L: 26.30 PLT, 10^9^/L: 599 HsCRP, mg/L: 157.2	Protozoa and helminths (-); Rotavirus (-); *Shigella* (-); *Salmonella* (-); Pathogenic *Escherichia coli* (-); *Plesiomonas* and *Aeromonas* (-)	No	TC, NAAT, Combined GDH and toxin EIA	Intravenous norvancomycin and oral norvancomycin, probiotics	Cured

Clinical history and laboratory tests are essential for the accurate diagnosis of CDI. This patient had diarrhea symptoms, abnormal stool routine examinations, and increased white blood cell count, CRP and muscle enzymes, which may be related to acute enteritis at the early stage of the disease. This clinical manifestation is difficult to distinguish from intestinal diseases caused by other predisposing factors. Therefore, the diagnosis of CDI in children remains an incredible clinical challenge. CDI is defined by the presence of symptoms (usually diarrhea) and either a stool test positive for *C. difficile* toxins or detection of toxigenic *C. difficile* or colonoscopic or histopathologic findings revealing pseudomembranous colitis [8]. However, no single laboratory test is considered the best. An immunoassay for GDH detects a highly conserved metabolic enzyme (common antigen) present in all isolates of *C. difficile.* GDH immunoassays cannot distinguish toxigenic *C. difficile* strains and lack specificity, so it is often used as a primary screening test for CDI [8]. TC has a high sensitivity (94–100%) and specificity (99%) which makes it a gold standard for laboratory diagnosis, but it has high experimental requirements and is not suitable for widespread use in clinical laboratories [41]. In addition, NAATs are capable of detecting the genes encoding *C. difficile* toxins A and B, making them an effective way to detect *C. difficile* [42]. However, when used in populations with high rates of *C. difficile* colonization, they may cause overdiagnosis of CDI due to their sensitivity [43]. Toxins in stool can distinguish colonization from infection more precisely, although recent studies have shown that asymptomatic children are more likely to have positive toxins in stool [44, 45]. The toxin EIA for *C. difficile* toxins A and/or B is inexpensive and easy to perform, but it is less sensitive than NAATs for detecting CDI and should not be used as a standalone test [8]. Consequently, the Infectious Diseases Society of America (IDSA) and the Society for Healthcare Epidemiology of America (SHEA) have provided some valuable recommendations for the laboratory diagnosis of *C. difficile* in adults and children [8]. To begin with, *C. difficile* testing should only be performed in patients with three or more unexplained unformed stools within 24 h. As a second recommendation, routine testing should be avoided in children under the age of 12 months unless other possible causes have been ruled out. Furthermore, ESCMID recommends multiple-step test for the accurate diagnosis of CDI [46]. The first test should be GDH assay or NAAT, which has a high negative predictive value. If the result is positive, the second assay should be highly specific, such as toxin EIA, which has a high positive predictive value. If the second test is positive, the final diagnosis is CDI. Patients with a negative second test for toxins should be re-evaluated (TC or NAATs) for the possibility of true infection. In this study, after excluding other potential causes, the infant was first tested for GDH combined with toxins by EIA, and the results showed GDH (+) and Toxins (-). Subsequently, to further evaluate whether the infant was truly infected, we performed TC and NAATs, and the results were positive. Finally, combined with the clinical symptoms, the patient was diagnosed with CDI.

Antibiotic therapy remains the first line of treatment of CDI, and antibiotics should be chosen according to guidelines and severity of the infection [41]. Currently, the treatment of CDI in children is based on clinical data from adults [47]. For children with their first episode or first recurrence of non-severe CDI, metronidazole and vancomycin are recommended. Oral vancomycin is preferred over metronidazole for children with a first episode of severe CDI [8]. Notably, new antibiotics may further disrupt intestinal microbiota, with approximately 25% of patients experiencing relapses within 4 weeks after antibiotic treatment [48]. FMT helps re-establish the intestinal microbiota and has a higher success rate than vancomycin when treating CDI that has relapsed/refractory [49]. Moreover, several studies have shown that probiotics reduce the incidence of antibiotic-associated diarrhea, and *Saccharomyces boulardii*, *Lactobacillus acidophilus*, *Lactobacillus casei*, and *Lactobacillus rhamnosus* can prevent primary or recurrent CDI [50–52]. In this case, after 3 days of intravenous norvancomycin (North China Pharmaceutical Company, China) according to the instructions, the frequency of diarrhea decreased. After continuing treatment with norvancomycin for 9 days, the infant’s stool frequency was normal, about 3 times/day, mainly viscous stools, and the condition was significantly improved. Stool culture of *C. difficile* and NAATs were still positive. After discharge, the patient was given oral norvancomycin and probiotic consolidation therapy. However, during this period, the patient had two relapses of diarrhea, accompanied by abnormal stool routine examination, *C. difficile* toxigenic culture, and NAATs were positive. Based on the clinical manifestations and laboratory diagnosis of the patient, the duration of the final treatment stage was extended, and the dose was adjusted in time according to the clinical status. Finally, her stool frequency returned to normal after three rounds of oral norvancomycin treatment. The patient had no recurrence of diarrhea, stool culture of *C. difficile*, GDH EIA, toxin EIA, and NAATs were negative during the 3-month follow-up period.

The ecology of the intestinal microbiota determines *C. difficile* colonization and virulence [53]. It has been reported that the alpha diversity of the intestinal microbiota decreased significantly in infants with NEC, and the abundance of *Bifidobacterium* and *Lactobacillus* decreased [54, 55]. The strictly anaerobes in the intestinal of patients with NEC are *Clostridium* species (*C. butyricum*, *C. neonatale*, *C. perfringens*, *C. paraputrificum*, and *C. difficile*), which are associated with NEC in preterm infants [56, 57]. Therefore, in our case, we hypothesized that the intestinal microbiota was altered by NEC and antibiotic therapy, facilitating the development of CDI. However, the relationship between NEC and CDI still needs to be further elucidated. On the other hand, the microbiota differs between caesarean section born and vaginally delivered infants over the first year of life, showing enrichment of *Enterococcus*, *Enterobacter*, *Clostridium perfringens* and *Klebsiella*, and reduction of *Bacteroides* and *Bifidobacterium* in caesarean section born infants [58–61]. Our patient was born by caesarian section. These data suggested that caesarian section may also be a risk factor for CDI in infants.

In the recovery of diseases, the intestinal microbiota plays an equally important role. Studies have shown that the abundance of *Firmicutes* and *Bacteroidetes* decreased while the abundance of *Proteobacteria* increased in children with toxin-positive *C. difficile* [62]. In the present study, the intestinal microbiota improved in the late stage of treatment and recovery period, demonstrated by the enrichment of *Firmicutes* and *Lactobacillus* and the increase in alpha diversity. *Lactobacillus* species, used as probiotics, regulate the intestinal microbiota, reduce intestinal inflammation, enhance host immune function, maintain the integrity of the intestinal barrier, and inhibit the production of toxins A and B [63, 64]. On the other hand, increased alpha diversity in the intestinal microbiota was also associated with age and complementary feeding [16, 65]. The effect of age and formula feeding in recovery was also considered during the study period. Further studies are necessary to determine the evolution and characteristics of the intestinal microbiota to better understand the relationship between *C. difficile*-associated dysbiosis and infant development.

In summary, based on the literature review and this case report, clinicians should also pay attention to diarrhea caused by *C. difficile* in infants and young children. More strong evidence is needed to explain the true prevalence of CDI in this population and to better understand the *C. difficile-*associated diarrhea in infants.

## Electronic supplementary material

Below is the link to the electronic supplementary material.

Supplementary Material 1: Table S1. Results of laboratory tests during the clinical course

## Data Availability

All data generated or analyzed during this study are included in this published article and its supplementary information files. The metagenomic sequences of intestinal microbiota are available in the NCBI Sequence Read Archive (SRA) (https://www.ncbi.nlm.nih.gov/sra) under BioProject accession number PRJNA961946.

## Abbreviations

CDI: *Clostridioides difficile* infection
FMT: Fecal microbiota transplantation
NEC: Necrotizing enterocolitis
CRP: C-reactive protein
HPF: High power field
EIA: Enzyme immunoassay
NAATs: Nucleic acid amplification tests
TC: Toxigenic culture
ASVs: Amplicon sequence variants
PCoA: Principal coordinate analysis
GDH: Glutamate dehydrogenase
IDSA: Infectious Diseases Society of America
SHEA: Society for Healthcare Epidemiology of America
WBC: White blood cells
PLT: Platelets
PCR: Polymerase chain reaction
ELISA: Enzyme-linked immunosorbent assay
CCNA: Cell culture cytotoxicity neutralization assay
RT-PCR: Real-time polymerase chain reaction
NA: Not available

## References

[1] Mileto S, Das A, Lyras D. Enterotoxic clostridia: *Clostridioides difficile* infections. MICROBIOL SPECTR. 2019;7. 10.1128/microbiolspec.gpp3-0015-2018 PMC11026080 31124432

[2] Miller BA, Chen LF, Sexton DJ, Anderson DJ (2011). Comparison of the burdens of hospital-onset, healthcare facility-associated *Clostridium difficile* infection and of healthcare-associated infection due to methicillin-resistant staphylococcus aureus in community hospitals. INFECT CONT HOSP EP.

[3] Janoir C (2016). Virulence factors of *Clostridium difficile* and their role during infection. Anaerobe.

[4] Fletcher JR, Pike CM, Parsons RJ, Rivera AJ, Foley MH, McLaren MR (2021). *Clostridioides difficile* exploits toxin-mediated inflammation to alter the host nutritional landscape and exclude competitors from the gut microbiota. NAT COMMUN.

[5] Peery AF, Dellon ES, Lund J, Crockett SD, McGowan CE, Bulsiewicz WJ (2012). Burden of gastrointestinal disease in the united states: 2012 update. Gastroenterology.

[6] Heimann SM, Cruz AM, Mellinghof S, Vehreschild M (2018). Economic burden and cost-effective management of *Clostridium difficile* infections. MED MALADIES INFECT.

[7] Honda H, Dubberke ER (2014). The changing epidemiology of *Clostridium difficile* infection. CURR OPIN GASTROEN.

[8] McDonald LC, Gerding DN, Johnson S, Bakken JS, Carroll KC, Coffin SE (2018). Clinical practice guidelines for *Clostridium difficile* infection in adults and children: 2017 update by the infectious diseases society of America (IDSA) and society for healthcare epidemiology of America (SHEA). CLIN INFECT DIS.

[9] Furuya-Kanamori L, Stone JC, Clark J, McKenzie SJ, Yakob L, Paterson DL (2015). Comorbidities, exposure to medications, and the risk of community-acquired *Clostridium difficile* infection: a systematic review and meta-analysis. INFECT CONT HOSP EP.

[10] Theriot CM, Young VB (2015). Interactions between the gastrointestinal microbiome and *Clostridium difficile*. ANNU REV MICROBIOL.

[11] Stoesser N, Eyre DW, Quan TP, Godwin H, Pill G, Mbuvi E (2017). Epidemiology of *Clostridium difficile* in infants in oxfordshire, uk: risk factors for colonization and carriage, and genetic overlap with regional c. difficile infection strains. PLoS ONE.

[12] Plants-Paris K, Bishoff D, Oyaro MO, Mwinyi B, Chappell C, Kituyi A (2019). Prevalence of *Clostridium difficile* infections among kenyan children with diarrhea. INT J INFECT DIS.

[13] Wang WP et al. Pediatrics: People’s Medical Press; 2018.

[14] Callahan BJ, McMurdie PJ, Rosen MJ, Han AW, Johnson AJ, Holmes SP (2016). Dada2: high-resolution sample inference from illumina amplicon data. NAT METHODS.

[15] Koljalg U, Nilsson RH, Abarenkov K, Tedersoo L, Taylor AF, Bahram M (2013). Towards a unified paradigm for sequence-based identification of fungi. MOL ECOL.

[16] Xiao L, Wang J, Zheng J, Li X, Zhao F (2021). Deterministic transition of enterotypes shapes the infant gut microbiome at an early age. GENOME BIOL.

[17] Chandrasekaran R, Lacy DB (2017). The role of toxins in *Clostridium difficile* infection. FEMS MICROBIOL REV.

[18] Pothoulakis C, Lamont JT (2001). Microbes and microbial toxins: paradigms for microbial-mucosal interactions ii. The integrated response of the intestine to *Clostridium difficile* toxins. AM J PHYSIOL-GASTR L.

[19] Holst E, Helin I, Mardh PA (1981). Recovery of *Clostridium difficile* from children. Scand J Infect Dis.

[20] Matsuki S, Ozaki E, Shozu M, Inoue M, Shimizu S, Yamaguchi N (2005). Colonization by *Clostridium difficile* of neonates in a hospital, and infants and children in three day-care facilities of kanazawa, japan. INT MICROBIOL.

[21] Jangi S, Lamont JT (2010). Asymptomatic colonization by *Clostridium difficile* in infants: implications for disease in later life. J PEDIATR GASTR NUTR.

[22] Kociolek LK, Espinosa RO, Gerding DN, Hauser AR, Ozer EA, Budz M (2020). Natural *clostridioides difficile* toxin immunization in colonized infants. CLIN INFECT DIS.

[23] Eglow R, Pothoulakis C, Itzkowitz S, Israel EJ, O’Keane CJ, Gong D (1992). Diminished *Clostridium difficile* toxin a sensitivity in newborn rabbit ileum is associated with decreased toxin a receptor. J CLIN INVEST.

[24] Kim J, Smathers SA, Prasad P, Leckerman KH, Coffin S, Zaoutis T (2008). Epidemiological features of *Clostridium difficile*associated disease among inpatients at children’s hospitals in the united states, 2001–2006. Pediatrics.

[25] Dong N, Li ZR, Qin P, Qiang CX, Yang J, Niu YN (2022). Risk factors for *Clostridioides difficile* infection in children: a systematic review and meta-analysis. J HOSP INFECT.

[26] Slimings C, Riley TV (2014). Antibiotics and hospital-acquired *Clostridium difficile* infection: update of systematic review and meta-analysis. J ANTIMICROB CHEMOTH.

[27] Khalil A, Hendaus MA, Elmagboul E, Mohamed A, Deshmukh A, Elmasoudi A (2019). Incidence of *Clostridium difficile* infection and associated risk factors among hospitalized children in qatar. THER CLIN RISK MANAG.

[28] Na JY, Park JM, Lee KS, Kang JO, Oh SH, Kim YJ (2014). Clinical characteristics of symptomatic *Clostridium difficile* infection in children: conditions as infection risks and whether probiotics is effective. PEDIATR GASTROENTERO.

[29] Kuntz JL, Chrischilles EA, Pendergast JF, Herwaldt LA, Polgreen PM (2011). Incidence of and risk factors for community-associated *Clostridium difficile* infection: a nested case-control study. BMC INFECT DIS.

[30] Cappella M, Pugliese F, Zucchini A, Marchetti F (2016). *Clostridium difficile* enterocolitis and reactive arthritis: a case report and review of the literature. CASE REP PEDIAT.

[31] Durand CL, Miller PF (2009). Severe *Clostridium difficile* colitis and reactive arthritis in a ten-year-old child. PEDIATR INFECT DIS J.

[32] Liang Y, He X, Wang T, Chen Y, Huang H, Tang W (2020). Massive hydrothorax and ascites as the primary manifestation of infection with *Clostridium difficile*: a case report and literature review. FRONT PEDIATR.

[33] Rojas GM, Jarasvaraparn C, Batten L, Custodio H, Gremse DA (2018). *Clostridium difficile* colitis complicating kawasaki disease in children: two case reports. SAGE OPEN MED CASE R.

[34] Price EH, Wright VM, Walker-Smith JA, Tabaqchali S (1988). *Clostridium difficile* and acute enterocolitis. ARCH DIS CHILD.

[35] Loffler HA, Pron B, Mouy R, Wulffraat NM, Prieur AM (2004). *Clostridium difficile*-associated reactive arthritis in two children. JOINT BONE SPINE.

[36] Nogueira H, Costa CL, Martins CS, Morais M, Quesada-Gomez C, Carvalho C (2021). Infection with *Clostridioides difficile* ribotype 046 in a paediatric liver transplant patient. Access Microbiol.

[37] Quesada-Gomez C, Vargas P, Lopez-Urena D, Gamboa-Coronado MM, Rodriguez-Cavallini E (2012). Community-acquired *Clostridium difficile* nap1/027-associated diarrhea in an eighteen month old child. Anaerobe.

[38] Yang Hong-bin, Ying FANG (2018). Ren Xiao-xia, HAN Ya-nan. *Clostridium difficile* enteritis in children: a case report. Chin J Practical Pediatr.

[39] Guan Jun HE, Lei-yan WANG, Yu-huan WANG, Chuan-qing HUANG, Ying YU, Hui (2017). Pseudomembranous enteritis caused by *Clostridium difficile* infection in a child: a case report. Chin J Evid Based Pediatr | Chin J Evid Based Pediatr.

[40] Kader A, O’Hare B, Valappil MK (2004). Non-antibiotic associated c. Difficile diarrhea in a 7 week-old infant. INDIAN PEDIATR.

[41] Napolitano LM, Edmiston CJ (2017). *Clostridium difficile* disease: diagnosis, pathogenesis, and treatment update. SURGERY.

[42] Antonara S, Leber AL (2016). Diagnosis of *Clostridium difficile* infections in children. J CLIN MICROBIOL.

[43] Luna RA, Boyanton BJ, Mehta S, Courtney EM, Webb CR, Revell PA (2011). Rapid stool-based diagnosis of *Clostridium difficile* infection by real-time pcr in a children’s hospital. J CLIN MICROBIOL.

[44] Polage CR, Gyorke CE, Kennedy MA, Leslie JL, Chin DL, Wang S (2015). Overdiagnosis of *Clostridium difficile* infection in the molecular test era. JAMA INTERN MED.

[45] Parnell JM, Fazili I, Bloch SC, Lacy DB, Garcia-Lopez VA, Bernard R (2021). Two-step testing for *Clostridioides difficile* is inadequate in differentiating infection from colonization in children. J PEDIATR GASTR NUTR.

[46] Crobach MJ, Planche T, Eckert C, Barbut F, Terveer EM, Dekkers OM (2016). European society of clinical microbiology and infectious diseases: update of the diagnostic guidance document for *Clostridium difficile* infection. CLIN MICROBIOL INFEC.

[47] Shim JO (2014). *Clostridium difficile* in children: to treat or not to treat?. PEDIATR GASTROENTERO.

[48] Louie TJ, Miller MA, Mullane KM, Weiss K, Lentnek A, Golan Y (2011). Fidaxomicin versus vancomycin for *Clostridium difficile* infection. NEW ENGL J MED.

[49] Gough E, Shaikh H, Manges AR (2011). Systematic review of intestinal microbiota transplantation (fecal bacteriotherapy) for recurrent *Clostridium difficile* infection. CLIN INFECT DIS.

[50] McFarland LV, Ship N, Auclair J, Millette M (2018). Primary prevention of *Clostridium difficile* infections with a specific probiotic combining lactobacillus acidophilus, l. Casei, and l. Rhamnosus strains: assessing the evidence. J HOSP INFECT.

[51] McFarland LV (2006). Meta-analysis of probiotics for the prevention of antibiotic associated diarrhea and the treatment of *Clostridium difficile* disease. AM J GASTROENTEROL.

[52] Hell M, Bernhofer C, Stalzer P, Kern JM, Claassen E (2013). Probiotics in *Clostridium difficile* infection: reviewing the need for a multistrain probiotic. BENEF MICROBES.

[53] Semon AK, Keenan O, Zackular JP (2021). *Clostridioides difficile* and the microbiota early in life. J PEDIAT INF DIS SOC.

[54] Ahearn-Ford S, Berrington JE, Stewart CJ. Development of the gut microbiome in early life. EXP PHYSIOL; 2022. 10.1113/EP089919 PMC9305283 35041771

[55] Duan M, Han Z, Huang N (2020). Changes of intestinal microflora in neonatal necrotizing enterocolitis: a single-center study. J INT MED RES.

[56] Schonherr-Hellec S, Klein GL, Delannoy J, Ferraris L, Roze JC, Butel MJ et al. Clostridial strain-specific characteristics associated with necrotizing enterocolitis. APPL ENVIRON MICROB. 2018;84. 10.1128/AEM.02428-17 PMC5861827 29352082

[57] Roze JC, Ancel PY, Lepage P, Martin-Marchand L, Al NZ, Delannoy J (2017). Nutritional strategies and gut microbiota composition as risk factors for necrotizing enterocolitis in very-preterm infants. AM J CLIN NUTR.

[58] Reyman M, van Houten MA, van Baarle D, Bosch A, Man WH, Chu M (2019). Impact of delivery mode-associated gut microbiota dynamics on health in the first year of life. NAT COMMUN.

[59] Shao Y, Forster SC, Tsaliki E, Vervier K, Strang A, Simpson N (2019). Stunted microbiota and opportunistic pathogen colonization in caesarean-section birth. Nature.

[60] Backhed F, Roswall J, Peng Y, Feng Q, Jia H, Kovatcheva-Datchary P (2015). Dynamics and stabilization of the human gut microbiome during the first year of life. CELL HOST MICROBE.

[61] Mueller NT, Differding MK, Ostbye T, Hoyo C, Benjamin-Neelon SE (2021). Association of birth mode of delivery with infant faecal microbiota, potential pathobionts, and short chain fatty acids: a longitudinal study over the first year of life. BJOG-INT J OBSTET GY.

[62] Lees EA, Carrol ED, Ellaby N, Roberts P, Corless CE, Lenzi L (2020). Characterization of circulating *Clostridium difficile* strains, host response and intestinal microbiome in hospitalized children with diarrhea. PEDIATR INFECT DIS J.

[63] Maziade PJ, Ship N, Sniffen JC, Goldstein E (2021). Enhanced *Clostridioides difficile* infection prevention with a pharmacy-controlled policy that adds a 3-strain lactobacillus probiotic concomitantly to antibiotic therapy. CLIN INFECT DIS.

[64] Malmqvist L, Ullberg M, Hed MI, Nilsson A (2019). *Clostridium difficile* infection in children: epidemiology and trend in a swedish tertiary care hospital. PEDIATR INFECT DIS J.

[65] Laursen MF. Gut microbiota development: influence of diet from infancy to toddlerhood. ANN NUTR METAB. 20211–14. 10.1159/000517912 34461613

