# Supplementary material for: Clostridioides difficile infection in infants: a case report and literature review
Source: Gut Pathog. 2023 Jun 29;15:31. doi: 10.1186/s13099-023-00552-1 (PMC10311876; doi:10.1186/s13099-023-00552-1)
Supplement: Supplementary file 1 — Supplementary Material 1: Table S1. Results of laboratory tests during the clinical course [file 13099_2023_552_MOESM1_ESM.docx]

**Supplementary Online Content**

**Table S1.** Results of laboratory tests during the clinical course

| **Variables** | **Hospitalization** | | | **Hospital discharge** | | | | |
| --- | --- | --- | --- | --- | --- | --- | --- | --- |
|  | **Hospital admission** | **Day 6** | **Day 11** | **After the first round of oral norvancomycin** | **After the first drug withdrawal** | **After the second round of oral norvancomycin** | **After the second drug withdrawal** | **After the third round of oral norvancomycin** |
| Red blood cell count, 10^12^/L (3.80−5.10) | 3.36 | 2.86 | 2.82 | - | - | - | - | 4.45 |
| White blood cell count, 10^9^/L (3.50−9.50) | 26.30 | 13.4 | 16.97 | - | - | - | - | 9.40 |
| Neutrophils, 10^9^/L (1.80−6.30) | 11.47 | 2.49 | 4.82 | - | - | - | - | 2.00 |
| Lymphocyte, 10^9^/L (1.10−3.20) | 6.87 | 7.26 | 7.28 | - | - | - | - | 6.43 |
| Monocyte, 10^9^/L (0.10−0.60) | 7.05 | 2.58 | 4.59 | - | - | - | - | 0.69 |
| Platelets, 10^9^/L (125−350) | 599 | 899 | 823 | - | - | - | - | 524 |
| Hemoglobin, g/L (110−120) | 104 | 86 | 84 | - | - | - | - | 121 |
| HsCRP, mg/L (0.00−6.00) | 157.2 | 50.3 | 53.1 | - | - | - | - | - |
| CK-MB, U/L (0.00−24.00) | 17 | - | 59 | - | - | - | - | - |
| LDH, U/L (120−250) | 355 | - | 256 | - | - | - | - | - |
| HBDH, U/L (72.00−182.00) | 302.9 | - | 199 | - | - | - | - | - |
| Albumin, g/L (40.00−55.00) | 31.4 | - | 31.6 | - | - | - | - | - |
| Prealbumin, g/L (0.18−0.35) | 0.05 | - | 0.06 | - | - | - | - | - |
| AST, U/L (13.00−35.00) | 37.8 | - | 31.5 | - | - | - | - | - |
| ALT, U/L (7.00−40.00) | - | - | 24 | - | - | - | - | - |
| GGT, U/L (7.00−45.00) | 327 | - | 214 | - | - | - | - | - |
| Procalcitonin, ng/mL | 0.2 | - | - | - | - | - | - | - |
| Creatinine, µmol/L (41−73) | - | - | - | - | - | - | - | - |
| Fecal red blood cell count, /HPF (0) | 15-25 | Normal | Normal | Normal | Normal | Normal | Normal | Normal |
| Fecal white blood cell count, /HPF (0-1) | 99-120 | 8-12 | Normal | 0-2 | 4-6 | Normal | 26-44 | Normal |

**Abbreviations:** HsCRP, high-sensitivity C-reactive protein; LDH, lactate dehydrogenase; HBDH, hydroxybutyrate dehydrogenase; AST, aspartate transaminase; ALT, alanine aminotransferase; GGT, γ-glutamyl transpeptidase; -, not available.
